# Surgically assisted rapid palatal expansion for transverse maxillary discrepancy in adults - Case report

**DOI:** 10.1016/j.ijscr.2021.106687

**Published:** 2021-12-21

**Authors:** Kanistika Jha, Manoj Adhikari

**Affiliations:** a Department of Orthodontics, College of Medical Sciences, Chitwan, Bharatpur, Nepal; b Department of Oral and Maxillofacial Surgery, Nepalese Army Institute of Health Sciences, College of Medicine, Sanobharyang, Kathmandu, Nepal

**Keywords:** Case report, SARPE, SARME, Distraction of maxilla, Constricted maxilla, Midpalatal split

## Abstract

**Introduction and importance:**

Transverse maxillary deficiency is one of the most detrimental problems to midfacial growth and the integrated dentoalveolar structures. Early diagnosis and proper treatment of such cases is most important to maintain the balance between the basal bones and stable occlusion.

**Case presentation:**

In our case, a 17-year-old male had irregular upper front teeth with an unpleasant smile. Detail examination revealed a symmetrical face with an orthognathic profile, mild malar deficiency, competent lips, asymmetrical arches, Class I molar and canine relationships bilaterally. Crowding was present in the upper anterior arch with 2 mm of anterior open bite and posterior cross bite present in the premolar region and molar region bilaterally. Lefort-1 osteotomy, midpalatal split, pterygomandibular disjunction without down fracture was done. The HYRAX appliance was cemented. Distraction started after four days of surgery. One mm distraction per day was done for 10 days. The patient was transferred to fixed orthodontic treatment to relive the anterior crowding. Records were taken after 1 year of follow up and analyzed. Skeletal relationships were in harmony. Dental crowding, anterior open bite and posterior crossbite were corrected.

**Clinical discussion:**

The zygomatic buttress and the pterygomaxillary junction are considered as the critical areas of resistance for maxillary expansion. Literature claims lefort-1 osteotomy in combination with palatal distraction results in more displacement and less stress in the maxilla.

**Conclusion:**

SARPE has proved to be clinically effective and stable for the correction of transversely deficient maxilla after cessation of growth in adult patients.

## Highlights

• Maxillary skeletal base is retrognathic but facial profile is orthognathic. But only transverse discrepancy was treated.
• Among orthognathic surgery, SARPE is rare and is indicated when only transverse discrepancy needs to be addressed.
• Orthognathic surgery is done in 3 stages. We did surgery-first approach here, thus only two stages of treatment was required.
• Soft tissue compensation is also taken into consideration along with position of skeletal base of maxilla.
• Complete orthodontic treatment requires about two years’ time but this SARPE case has been completed in one year.

## 1 Introduction

Transverse maxillary deficiency is one of the most detrimental problems to mid facial growth and the integrated dentoalveolar structures [1]. Early diagnosis and proper treatment of such cases is most important to maintain the balance between the basal bones and stable occlusion. The management of maxillary transverse deficiency depends upon the severity and age/sex factors. According to literature, opening of the mid palatal suture during growth spurt can be achieved with rapid palatal expansion [2], while in adult cases, palatal resistance to expansion increases, leading to dentoalveolar expansion, buccal tipping of dentition, posterior extrusion, lateral compression of the periodontal membrane, buccal root resorption, palatal tissue necrosis, and fenestration in the buccal cortex instead of an increase in basal width of the maxilla.

Slow and rapid maxillary expansion is modalities for correction of transverse maxillary deficiency. In mild to moderate adult cases, (discrepancy less than 5 mm) this can be treated by slow maxillary expansion. However, maxillary transverse dysplasia of greater than 5 mm needs to be treated using various osteotomies protocols for true skeletal expansion [3]. Lefort-1 osteotomy to free the maxilla from all the cranial bones to gain a true increase in maxillary width or partial osteotomy with attachment of expanders (SARPE) to reduce resistance for rapid expanders can be treatment options in adult cases.

Most of the cases of skeletal Class III malocclusion require three-piece maxilla for correction of all anteroposterior, vertical and transverse discrepancy. But this case is specific as being skeletal Class III due to retrognathic maxilla with transverse discrepancy, the profile is orthognathic, soft tissue is in harmony with skeletal bases. From investigation and diagnosis, we concluded that space is required to align the crowding along with correction of transverse discrepancy with no change in facial profile. Therefore, only SARPE was considered in this case instead of three-piece maxilla. This case was managed at Nepalese Army Institute of Health Sciences, college of Medicine, tertiary center, Kathmandu, Nepal. This case of SARPE is presented here according to SCARE 2020 guidelines [4].

## 2 Presentation of case

**Fig. 1:**
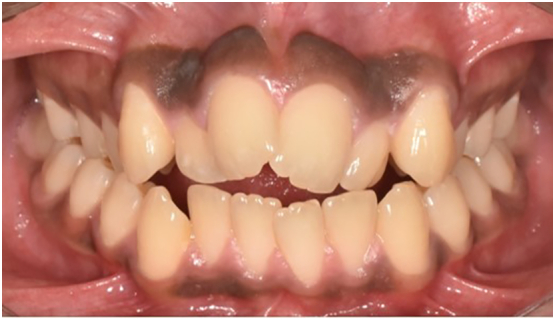
Pre-treatment intraoral frontal view of occlusion.

**Fig. 2:**
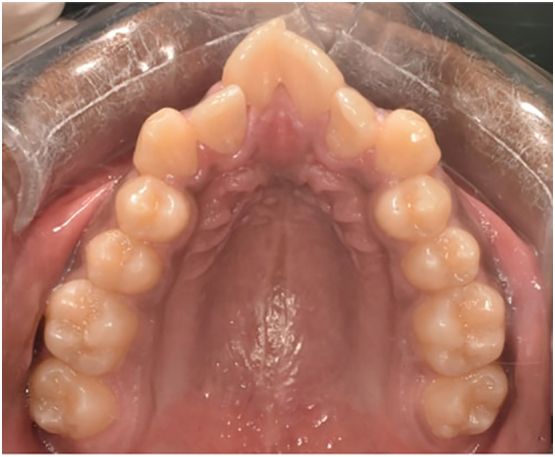
Pre-treatment intraoral maxillary occlusal photographs.

A 17-year-old male reported to the dental department with complaints of irregularly placed upper front teeth and an unpleasant smile without any significant medical or dental history. There was no any relevant drug history, family history, psychosocial history or any relevant genetic information. Pretreatment records were taken and analyzed. Extra-oral examinations revealed a symmetrical face with an orthognathic profile, mild malar deficiency with competent lips. Intraoral examination revealed asymmetrical arches with Class I molar and canine relationships bilaterally. Crowding is present in the upper anterior arch with 2 mm of anterior open bite and posterior cross bite present in the premolar and molar region bilaterally (Fig. 1, Fig. 2).

Lateral cephalogram analysis confirmed Class III skeletal base relationship (ANB: −3.0°, Wits appraisal: −3.2 mm, SNA: 76.7°, SNB: 79.3°) with a vertical growth pattern (SN-MP: 38.2° FMA-30°, Jarabak ratio - 61.26%). The inclination of the maxillary and mandibular incisors were within normal range (U1-SN: 112.3°, U1-NA: 30°, IMPA: 96°). The patient had an average nasolabial angle (96.3°), and his lower lip was protrusive relative to the E line (+1 mm).

Posteroanterior cephalogram (PA) analysis revealed symmetrical facial skeleton with constricted maxillary arch (J-J′: 58 mm, it should be 68 mm at 16 years of age). The maxilla-mandibular differential index was 72.5% (Normal 80%) suggestive of maxillary constriction.

The dental cast analysis showed discrepancy of 12 mm with an inter-canine width of 33 mm, an inter-premolar width of 36 mm and an inter-molar width of 47 mm in the maxillary arch.

## 3 Treatment objectives

Based on chief complain and diagnostic records the following treatment objectives were derived. 1. To expand the maxillary base. 2. To achieve alignment and leveling 3. To achieve normal inclination and placement of upper and lower anterior. 4. To maintain normal overjet and overbite. 5. To maintain the facial profile.

## 4 Treatment planning

Ideal treatment planning was orthognathic surgery involving Phase I (surgery)-Patient treated with SARPE (Surgically Assisted Rapid Palatal Expansion). Phase II –alignment and leveling.

The patient was scheduled for SARPE. The bonded HYRAX appliance was constructed. A day before surgery, an appliance trial was conducted. The appliance was checked for proper fitness and any discomfort.

## 5 Surgery

**Fig. 3:**
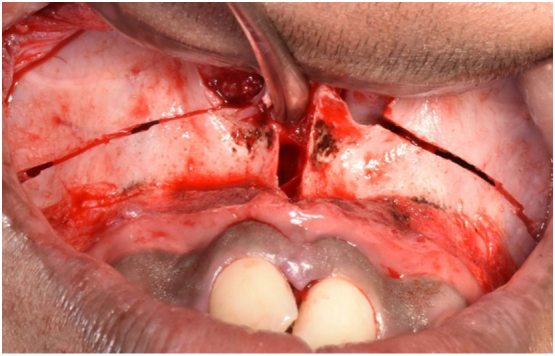
Intraoperative photograph showing surgical cuts at lefort-1 level and mid-palatal split.

The patient was operated under general anesthesia. Orthognathic surgery was done by consultant Oral and Maxillofacial surgeon, Dr. Manoj Adhikari. The incision site was marked in the maxillary vestibule, 6 mm above the mucogingival junction, extending from maxillary left 1st molar tooth to right 1st molar. 2% lignocaine with adrenaline (1:2 lakh dilution) was injected at incision site. The incision was given, mucoperiosteal flap was raised. An osteotomy cut was given using piezotome extending from inferolateral margin of anterior nasal aperture to posterolateral area of maxilla bilaterally. Lateral nasal osteotomy was done using lateral nasal osteotome. Septal osteotomy was done using nasal septal osteotome. Pterygomaxillary disjunction was done with a curved chisel and mallet bilaterally. Down fracture was not done. Palatal osteotomy was done with piezotome, starting anteriorly between two central incisors (Fig. 3). Prefabricated Hyrax was cemented and few turns were given to confirm completeness of palatal osteotomy. After confirmation turn was reversed. Flap closure was done using standard technique. Surgery was completed without any complications. All the post-surgical instructions were given and analgesics along with antibiotics were prescribed to the patient.

## 6 Treatment progress

After 4 days of surgery, expansion was started with 2 turns/day (1 mm opening per day). Expansion continued for 10 days and a total of 8 mm of expansion was achieved. A midline diastema of 8 mm appeared and expansion was stopped. Records were taken to evaluate the midpalatal opening and required expansion achieved. The expansion appliance with a rigid framework was maintained in its place for one year. After six months, the patient was transferred to fixed orthodontic treatment to relive the anterior crowding. Upper full arch bonding was done and sequential wire was progressed to align the upper/lower arches. Slowly, the midline diastema started to close. Occlusion settling was achieved. Orthodontic treatment was done by consultant Orthodontics, Dr. Kanistika Jha. Records were taken again after one year of follow up and analyzed.

## 7 Treatment results

**Fig. 4:**
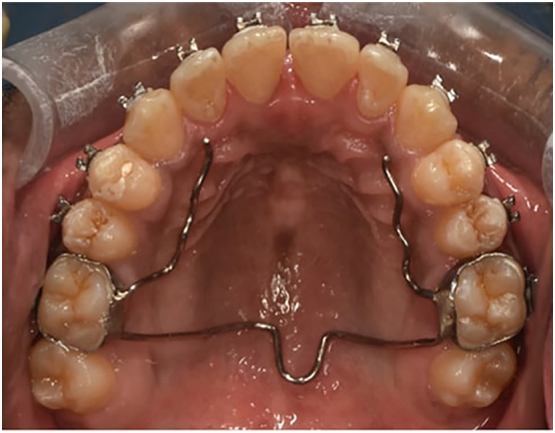
Post-treatment intraoral maxillary occlusal photograph with brackets and retainer in situ.

**Fig. 5:**
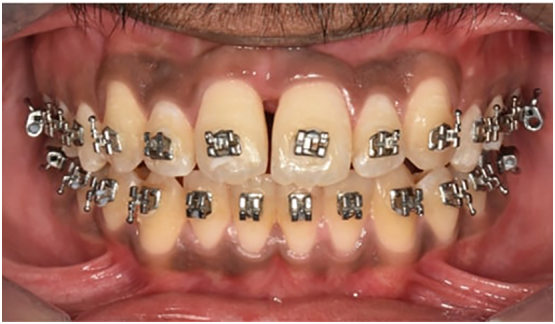
Post-treatment intraoral frontal view of occlusion.

**Supplementary Fig. 6:**
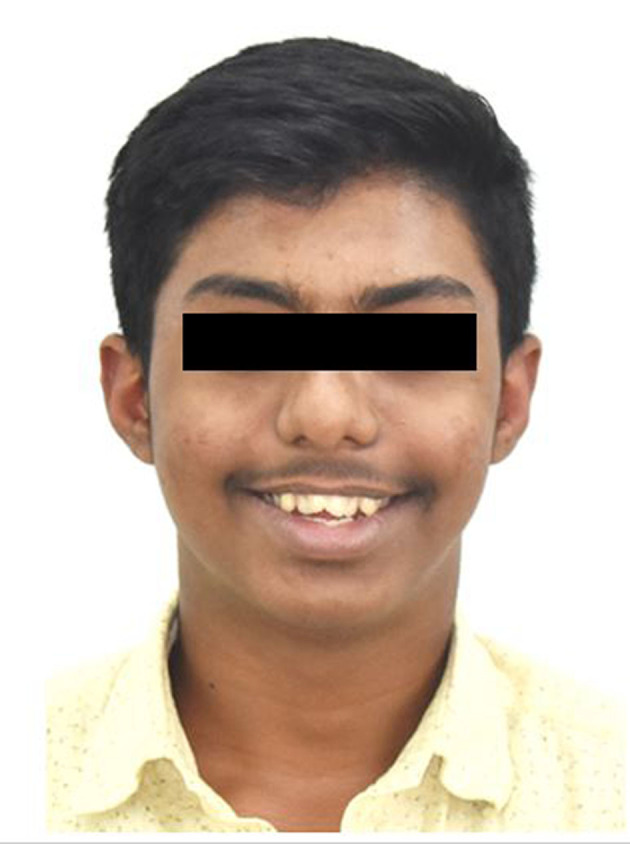
Pre-operative frontal view of patient in smile showing malaligned teeth.

**Supplementary Fig. 7:**
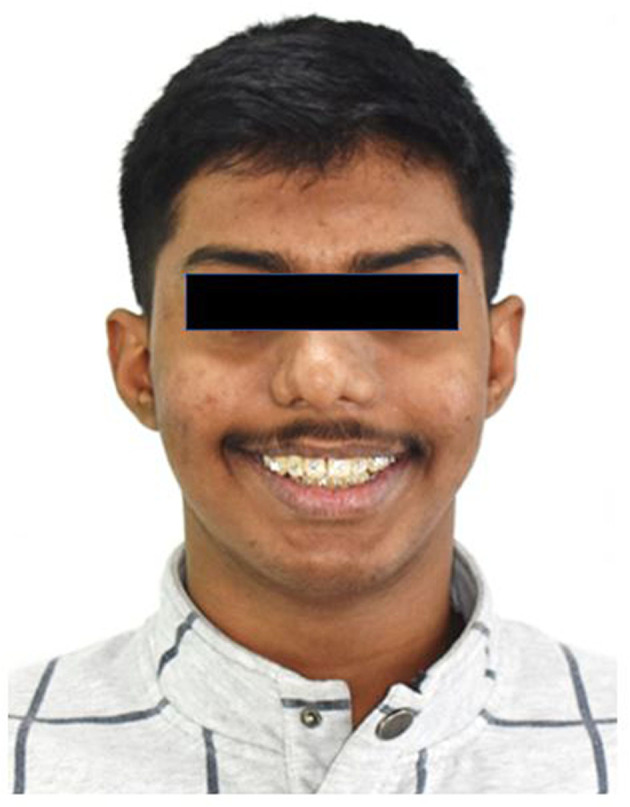
Post-operative frontal view of patient in smile showing well aligned teeth.

The treatment outcome was satisfactory. Skeletal relationships were in harmony. Dental crowding, anterior open bite and posterior crossbite were corrected (Figs. 4 and 5). Preoperative and Postoperative frontal view of face is shown in Figure Supplementary Figure 6 and Figure Supplementary Figure 7 respectively. No periodontal complications like gingival recession or bony dehiscence were observed after treatment. Post expansion study model analysis and posteroanterior cephalometry evaluation were done. Model analysis reveals net expansion of 4 mm in canine region, 5 mm in first premolar region and 6 mm in first molar region (Table 1). Cephalogram reveals maxillary skeletal base width increases by 5 mm (Table 2).

**Table 1:** Mean interdental distance measured on models (mm).

variables	Pre expansion (T1 in mm)	Post expansion (T2 in mm)	One year post expansion (T3 in mm)	T3-T1 (difference from start of treatment in mm)
Canines (mm)	33	38	37	4
First premolars (mm)	36	42	41	5
First molars (mm)	47	55	53	6

**Table 2:** Posteroanterior cephalogram analysis.

Variables	Pre expansion (T1)	Post expansion (T2)	T2-T1	One year post expansion (T3)	T3-T1 (difference from start of treatment
Maxillary Width -J′-J′ (mm)	58 mm	64 mm	6 mm	63 mm	5 mm

## 8 Discussion

The aim of this study was to manage adult cases of transverse maxillary dysplasia surgically with maxillary osteotomies procedures. Though we have different approaches to manage transverse dysplasia, ossified maxillary sutures and periodontal complications are the great hindrance. To avoid the complications SARPE was considered as treatment plan [5]. The case was started with orthognathic surgery followed by orthodontic treatment, i.e., two-step procedures rather than the conventional three-step surgical orthodontic approach, including presurgical orthodontic, orthognathic surgery, and postoperative orthodontic was done. The long duration of treatment and negative compliance of the patient in the three-step technique were taken into consideration.

The zygomatic buttress and the pterygo-maxillary junction are considered as the critical areas of resistance for maxillary expansion. Therefore, subtotal lefort-1 osteotomy, paramedian split, along with palatal distraction, is a widely used surgical technique [6]. Literature claims lefort-1 osteotomy in combination with palatal distraction results in more displacement and less stress in the maxilla [7]. During surgery, an osteotomy cut is given along all the lateral support of the maxilla followed by a mid-palatal split. For palatal distraction, the tooth-borne appliance HYRAX was used. Recent randomized controlled trial (RCT) has shown comparable effects of both tooth-borne and bone borne distractors [8]. Bonded HYRAX has shown beneficiary effects over vertical control as compared to banded HYRAX [9]. The distraction started 4 days after surgery once the callus bone was formed and the rate of expansion was 2 turns per day (1 mm per day). The literature is unclear about the activation schedule, so the rate of distraction osteogenesis has been recommended in this study.

The total amount of expansion in first molar region was 6 mm one year after retention, which corresponds with previous findings (Berger et al [10]). A total relapse of about 4% was measured one year post retention period. Inter-canine and inter-premolar expansion were 4 mm and 5 mm in one year post retention. Less relapse in this study was because the same HYRAX with a rigid framework was used till one year post retention. On PA cephalometric evaluation, skeletal maxillary expansion was 5 mm (J′-J′), which is coincident with Berger et al. [10]

## 9 Conclusion

Over recent years, SARPE has proved to be clinically effective and stable for the correction of transversely deficient maxilla. Surgery first approach with only two stage treatment, instead of traditional three stage treatment, can be done for SARPE. While making the treatment plan, soft tissue must be taken into consideration along with underlying bone.

## Patient perspective

The patient was very happy and grateful to the operating team. His appearance was completely changed, treatment duration was also short and did not have any complications.

## Source of funding

This research did not receive any specific grant from funding agencies in the public, commercial, or not-for-profit sectors.

## Ethical approval

It is our routine standard surgical procedure so ethical clearance was not required.

## Consent

Written informed consent was obtained from the patient for publication of this case report and accompanying images. A copy of the written consent is available for review by the Editor-in-Chief of this journal on request.

## Author contribution

Dr. Kanistika Jha: First Author.

Contributions: study concept or design, data collection, data analysis or interpretation, writing the paper, orthodontic treatment etc.

Dr. Manoj Adhikari: Corresponding Author and Co-Author.

Contributions: study concept or design, data collection, data analysis or interpretation, writing the paper, surgical treatment etc.

## Research registration

This case report does not include any ‘first in man’ studies, so, registration was not required.

## Guarantor

Dr. Manoj Adhikari, Consultant Oral and Maxillofacial Surgeon, Nepalese Army Institute of Health Sciences, College of Medicine, Kathmandu, Nepal.

## Provenance and peer review

Not commissioned, externally peer-reviewed.

## Declaration of competing interest

The authors declare that there is no conflict of interests regarding the publication of this paper.
